# Anti-Biofilm Property of Bioactive Upconversion Nanocomposites Containing Chlorin e6 against Periodontal Pathogens

**DOI:** 10.3390/molecules24152692

**Published:** 2019-07-24

**Authors:** Tianshou Zhang, Di Ying, Manlin Qi, Xue Li, Li Fu, Xiaolin Sun, Lin Wang, Yanmin Zhou

**Affiliations:** 1 Department of Oral Implantology, School of Stomatology, Jilin University, Changchun 130021, China; 2 Jilin Provincial Key Laboratory of Sciences and Technology for Stomatology Nanoengineering, Changchun 130021, China; 3 Department of Geriatric Dentistry, School of Stomatology, Jilin University, Changchun 130021, China

**Keywords:** upconversion nanoparticles, antibacterial photodynamic therapy, photosensitizer, manganese ion, periodontitis

## Abstract

Photodynamic therapy (PDT) based periodontal disease treatment has received extensive attention. However, the deep tissue location of periodontal plaque makes the conventional PDT encounter a bottleneck. Herein, upconversion fluorescent nanomaterial with near-infrared light excitation was introduced into the treatment of periodontal disease, overcoming the limited tissue penetration depth of visible light in PDT. Photosensitizer Ce6 molecules were combined with upconversion nanoparticles (UCNPs) NaYF_4_:Yb,Er with a novel strategy. The hydrophobic UCNPs were modified with amphiphilic silane, utilizing the hydrophobic chain of the silane to bind to the hydrophobic groups of the UCNPs through a hydrophobic-hydrophobic interaction, and the Ce6 molecules were loaded in this hydrophobic layer. This achieves both the conversion of the hydrophobic to the hydrophilic surface and the loading of the oily photosensitizer molecules. Because the excitation position of the Ce6 molecule is in the red region, Mn ions were doped to enhance red light, and thus the improved PDT function. This Ce6 loaded UCNPs composites with efficient red upconversion luminescence show remarkable bacteriological therapeutic effect on *Porphyromonas gingivalis*, *Prevotella intermedia* and *Fusobacterium nucleatum* and the corresponding biofilms under 980 nm irradiation, indicating a high application prospect in the treatment of periodontal diseases.

## 1. Introduction

With the changes in human living habits, periodontal disease has become the "top chronic killer" in oral diseases [1]. Periodontal treatment includes surgical and non-surgical methods. Underarm treatment and root smoothing can provide more recovery of the operation area, which, however, may also cause the additional risks of bleeding, pain, and infection in the patients’ gums during the treatments, increasing patient discomfort [2]. In recent years, non-surgical methods have attracted more and more attention, with an aim to avoid the above problems in controlling and treating periodontal disease. Photodynamic therapy is one of the most important non-surgical treatment methods, combining special photosensitizers with bio-optical techniques. This phototherapy method in dentistry, also known as antimicrobial PDT (aPDT), displays efficient bactericidal performance for oral pathogens [3]. With the emergence of drug-resistant strains due to the abuse of antibiotics and bacterial mutations, aPDT becomes especially important. The basic principles of photodynamic therapy are as follows: After the applied photosensitizer is attached to the bacteria, the laser light is introduced and the photosensitizer absorbs light energy into the singlet state from the stable triplet state. The singlet photosensitizer is extremely unstable and will instantaneously release the energy, returning to a triplet state [4]. The released energy is absorbed by tissue oxygen, forming reactive oxygen species (ROS) which have strong oxidation and high reactivity, causing the rapid lipid oxidation of bacteria, especially the destruction of vulnerable membrane lipids, and eventually bacterial death [5].

Antimicrobial PDT has been considered to be an important method in killing inflammation-related pathogens around teeth. Hass et al. firstly conducted the in vitro study on this point [6]. Three bacteria, *Prevotella intermedia* (*P. intermedia*), *Aggregatibacter actimycetemcomitans* (*A. actimycetemcomitans)*, *Porphyromonas gingivalis* (*P. gingivalis*), were selected and treated with aPDT method, respectively. All above three species were completely killed, confirming the high efficacy of bacteria inhibition compared with other treatment methods [6]. Chan et al. used methylene blue (MB) as a photosensitizer to kill three kinds of bacteria, *P. gingivalis*, A. *actimycetemcomitans* and *Fusobacterium nucleatum* (*F. nucleatum*) with inhibition rate of 95% [7]. Ayano *et al*. investigated the aPDT effect on *P. gingivalis* with photosensitizer rose bengal (RB) and the results show that aPDT from RB can effectively kill *P. gingivalis* with blue light [8]. Goulart et al. found *A. actimycetemcomitans* can be effectively inhibited with MB under the irradiation of light from 400 to 500 nm, and the number of surviving bacteria was significantly reduced [9]. Eick et al. investigated the aPDT from the toluidine blue (TB) with light excitation (625–635 nm), which can significantly reduce *A. actimycetemcomitans* biofilm activity formed by two different strains [10]. Street et al. combined the MB and lasers (650 to 675 nm) and found more effective treatment effect on *F. nucleatum* planktonic bacteria than that of biofilms, indicating that the effect of PDT on bacteria is also related to the bacteria form [11].

Although photodynamic therapy has made important progress in the treatment of periodontal disease, it is still in its primary stage, and there are some serious problems that are still necessary to be solved before clinical application. The most important one of conventional photodynamic therapy is the weak tissue penetration of ultraviolet or visible light. Therefore, it is highly desirable to design and prepare a photodynamic therapy system with the infrared irradiation light, which can penetrate deep tissues.

Upconversion fluorescence is an anti-stokes process that allows the long excitation wavelength to be converted to short emission wavelength. Rare earth doped upconversion nanoparticles (UCNPs) are the most outstanding representatives which can convert the infrared light to visible emission light by the continuous two-photon or multiphoton energy transfer process [12,13,14]. Upconversion brings many advantages: First, there is zero noise in biological background because biological tissue does not emit light under near-infrared (NIR) light, resulting in high signal-to-noise ratio in applications [15]. Second, the NIR excitation light used for up-conversion luminescence is located in the optical imaging window of the biological tissue, and has a deep tissue penetration. Red light can penetrate inside the tissue for 0.5 cm, while 980 nm light can penetrate more than 1 cm [16]. Third, some other advantages should be mentioned, such as narrow emission band, high color purity and stability, low toxicity and no photobleaching [17,18]. Therefore, if the up-conversion material were introduced in the periodontal deep tissue, the NIR light can be converted into light in different wavelengths from ultraviolet to NIR, satisfying the need to excite photosensitizers with different absorption bands, and well compensating for the low penetration of conventional photodynamic therapy light sources.

Note that the PDT triggered by upconversion light was first designed and applied in tumor therapy [19]. However, until now there have been no reports about such upconversion nanomaterials-based photodynamic therapy on periodontitis treatment, which is highly expected to be achieved if non-invasive periodontitis treatment is considered.

In this work, we designed a new UCNP/Ce6 composite nanomaterial with enhanced red light emission and efficient aPDT bactericidal performance. The combination of Ce6 and NaYF_4_:Yb,Er UCNPs was realized by using the amphiphilic silane modification technique, which involved the hydrophobic-hydrophobic interaction between hydrophobic side chain of the silane and hydrophobic groups on the surface of UCNPs [20]. In the NaYF_4_:Yb,Er/Ce6 composites, after hydrolysis, the silane forms a very thin hydrophilic coating, which means the hydrophobic UCNPs were converted to hydrophilic ones without increasing size and influencing the luminescent centers on the surface of UCNPs, since the hydrophobic side chain of the silane does not touch the surface of UCNPs. In addition, this design successfully avoids the leakage problem in conventional photosensitizer carriers by physical adsorption, such as photosensitizer loading in mesopores UCNP@mSiO_2_ NPs [19], which may lead to serious leakage problems, causing systemic toxic side effects as well. As the PDT function of Ce6 molecule should be triggered by excitation of red light, Mn doping is involved in this work, which greatly improves the probability of the red emission transition [18,21]. As a result, the enhanced upconversion red emission greatly improved the PDT effect on periodontal disease. The Ce6 loaded UCNPs composites with efficient red upconversion luminescence could be potentially employed for aPDT applications.

## 2. Experiments

### 2.1. Preparation of NaYF_4_:Yb^3+^,Er^3+^-Mn UCNPs

All experiments NaYF_4_:Yb^3+^,Er^3+^ UCNPs were synthesized by a high temperature thermal decomposition method. The specific experimental steps are as follows: 2.5 mmol NaOH and 4 mmol NH_4_F were dissolved in the 5 mL of methanol.0.78 mmol YCl_3_•6H_2_O, 0.20 mmol YbCl_3_•6H_2_O and 0.02 mmol ErCl_3_•6H_2_O were added in a three-necked flask and dissolved in 15 mL octadecene and 6 mL oleic acid and stirred. After the contents in the three-necked flask were dissolved at 80 °C, the whole three-necked flask was cooled to 30 °C, and the methanol solution was added dropwise, and the mixed solution was magnetically stirred for 30 min. Then the solution was heated to 125 °C and the methanol solution was evaporated. After the complete evaporation of methanol, it was heated to 320 °C under nitrogen for one hour. When the temperature of the solution was cooled to room temperature, the product was washed with a 1/1 ratio of ethanol and deionized water, and the final sample was obtained. Note that during the reaction those products are rapidly oxidized by oxygen in the air, and the desired sample cannot be prepared. Therefore, nitrogen is used as an inert gas, which is very necessary for protection. For the NaYF_4_:Yb^3+^,Er^3+^,Mn^2+^ UCNPs, the corresponding MnCl_2_ were added together with lanthanide elements, and the doping ratio of Mn is 0%, 10%, 20%, 30%, respectively [21].

### 2.2. Preparation of NaYF_4_:Yb^3+^,Er^3+^@Ce6@Silane Composites with and without Mn Doping

The amphiphilic silane has a hydrophobic alkane chain with a chain length of 18 carbon atoms. The silane was first dissolved in tetrahydrofuran solvent according to a mass ratio of silane/NaYF_4_=1/2. After that, Ce6 molecules were added to the mixed solution of NaYF_4_:Yb^3+^,Er^3+^ and silane in a certain ratio, and mixed under ultrasonic conditions for half an hour. Then the mixed solution was quickly injected into ammonia water with a pH of 9. After 3 hours of the hydrolysis reaction, it was transferred into a dialysis bag (molecular weight cutoff of 8000–14,000) for dialysis. The resulting upconversion composite not only has the function of up-converting fluorescent labeling, but also has PDT function. Mn doped UCNPs followed the same modification process with Ce6 and silane.

### 2.3. Characterization of NaYF_4_:Yb^3+^,Er^3+^@Ce6@Silane Composites with and without Mn Doping

The XRD measurement of the prepared samples was carried out using a Rigaku D/max 2550 X-ray diffractometer with a test angle of 15 to 80 degrees; the morphology of the synthesized samples was measured by a transmission electron microscope (TEM) of Hitachi H-800 (Hitachi, Ltd., Tokyo, Japan) with an operating voltage of 200 kV; The Fourier infrared (FTIR) spectra Shimadzu DT-40 model 883 IR spectrophotometer (Shimadzu Co., Kyoto, Japan) was used to measure the organic groups on the surface of UCNPs. For the specific operation process, 3 mL of the samples (UCNPs and UCNPs@silane at an approximate concentration of 4 μg/mL) was taken to be tested with the addition of potassium bromide powder, placed in an oven at 60 degrees Celsius, and the samples were dried for two days until the liquid completely disappeared. The Zeta potential of the UCNPs was determined by Zeta potential instrument (Zetasizer, Nano-Z, Malvern Instruments Limited, UK). The absorption spectrum of the sample was tested by UV-visible spectrophotometer Shimadzu UV-1800 (Shimadzu Co., Kyoto, Japan); SENS-9000 spectrometer (Zolix Instruments Co. Ltd., Beijing, China) is used to measure the excitation and emission spectra of the samples. The Andor Shamrock SR-750 spectrometer (Andor Technology Ltd., Tokyo, Japan) was used to test the up-conversion spectra of nanoparticles with a continuous 980 nm diode laser, and the upconversion signals were collected by photomultiplier tubes and monochromators. The detection range was set from 300 nm to 750 nm.

### 2.4. Dark Cell Toxicity of Mn-Doped UCNPs

L929 mouse fibroblasts were selected to detect dark cytotoxicity. The cells were obtained from the Institute of Biochemistry and Cell Biology of the Chinese Academy of Sciences (Shanghai, China) and approved by the Institutional Review Board of the Jilin University School of Dentistry. Fibroblasts were cultured in DMEM medium (HyClone, Logan, UT, USA) containing 1% antibiotics (100 U/mL penicillin and 100 μg/mL streptomycin) and 10% fetal bovine serum (Gibco, Carlsbad, CA, USA). The culture temperature of the cells was 37 °C, and the atmosphere contains 5% CO_2_. For the dark cytotoxicity of NaYF_4_:Yb^3+^,Er^3+^@Ce6@silane, L929 cells were incubated in 96-well plate at a density 5000 cells per well for 24 h. Then the cells were incubated with different concentration of NaYF_4_:Yb^3+^,Er^3+^@Ce6@silane for another 24 h in darkness. The cell viability was determined by CCK-8 assay (7sea biotech Ltd., Shanghai, China) following the instructions. Wells without any nanoparticles served as calibrators. The percentage survival was calculated and based on the control sample without any treatment as being 100%. All measurements were tested in three replicates.

### 2.5. Bacterial Culture

All three bacterial species were purchased from the American Type Culture Collection (ATCC, Manassas, VA): *Porphyromonas gingivalis* (*P. gingivalis*, ATCC 33277), *Prevotella intermedia* (*P. intermedia*, ATCC) and *Fusobacterium nucleatum* (*F. nucleatum*, ATCC 25586). The use of bacterial species was approved by the Institutional Review Board of the Jilin University School of Dentistry. All bacteria were cultured under anaerobic conditions of 80% N_2_, 10% H_2_ and 10% CO_2_ cultured with tryptic soy broth (TSB, Sigma-Aldrich, St. Louis, MO, USA) supplemented with vitamin K (1 mg/L), L-cysteine hydrochloride (0.5 g/L, Sigma-Aldrich), yeast extract (5 g/L, Sigma-Aldrich) and hemin (5 mg/L, Sigma-Aldrich).

### 2.6. aPDT of NaYF_4_@Ce@Silane Against Biofilm Formation On Dentin Squares

Caries-free human molars were extracted for the preparation of dentin samples, which was approved by the Institutional Review Board of the Jilin University School of Dentistry (Ref. H20170062), serving as the substrates for biofilm formation.

A square dentin sample of 5 × 5 mm (thickness of about 1 mm) was prepared and ground with silicon carbide paper, and then sterilized by autoclaving (134 °C, 15 min). Before every experiment, UV light for 30 minutes was involved and followed by immersion in saliva at 37 °C for 2 h to pre-coat the saliva film. A saliva sample was prepared from the mixed saliva from fifteen healthy donors who had not taken any antibiotics for 3 months. The saliva from the donors follows the requirements that there was no teeth brushing for 24 h and no food/beverage for 2 h before donating. Cell debris was removed from saliva by centrifugation of 3000 rpm for 20 min. A sterile 0.22 μm filter was used to filtrate the supernatant for sterilization (VWR International, Radnor, PA, USA). Salivary pellicle on dentin disks was prepared by immersing the disk in sterile saliva (37 °C) [22].

Three single-strain biofilms were prepared with each bacteria species [22]. Each species was inoculated (10^8^ CFU/mL) onto a salivary pellicle-coated dentin square in a 24-well plate. Then NaYF_4_@Ce@silane and Mn doped UCNPs were used to treat the samples with a concentration of 100 μg/mL. Then, it was irradiated with a 980 nm laser at 750 J·cm^−2^ for 3 min. The medium containing NaYF_4_@Ce@silane NPs was renewed every 24 h. The dentin square with the adhered biofilm was transferred to a new 24-well plate and treated with 980 nm light irradiation in the same way.

A mixture of SYTO 9 (2.5 μM, Invitrogen, Carlsbad, California) and propidium iodide (2.5 μM, Invitrogen, Carlsbad, California) was used to stain each sample for live/dead bacterial analysis (Molecular Probes, Eugene, OR, USA) [23]. A confocal laser scanning microscope (CLSM) was employed for the biofilms imaging. All experiments were conducted three times. Five images were randomly selected from each sample. For the three bacterial species, 15 images per group were obtained.

For the five groups and three bacterial species, a 5 × 3 full factorial design was used for CFU counting. Biofilms were collected from the samples by mechanical scraping. Then the corresponding bacterial deposits and samples were transferred to vials with 2 mL cysteine peptone water (CPW) and dispersed with ultrasound. Diluted suspensions of the biofilm were streaked onto Columbia blood agar and cultured at 37 °C for 48 h under anaerobic conditions of 80% N_2_, 10% H_2_ and 10% CO_2_ and then the CFU was counted, along with the dilution factor [24].

### 2.7. Polysaccharide Production of 4-day Biofilms

The water-insoluble polysaccharide of the biofilm of (A) *P. gingivalis*, (B) *P. intermedia* and (C) *F. nucleatum* on dentin squares was investigated via a phenol-sulfuric acid method [23]. The 4-day biofilms were immersed in a vial with 2 mL CPW and then collected by sonication/votexing [23]. A precipitate was obtained by centrifugation and then rinsed twice with PBS and resuspended in 1 mL of de-ionized water. After that, the solution was added to 1 mL of a 6% phenol into the vial, followed by 5mL of 95–97% sulfuric acid and incubated at 25 °C for half an hour. Then, 100 μL of the above solution was transferred into a 96-well plate. The amount of polysaccharide in the biofilm can be measured by the absorbance at 490 nm with the microplate reader (SpectraMax M5, Sunnyvale, CA, USA). The standard was made with five glucose concentrations of 0, 5, 10, 20, 50 and 100 mg/L in the conversion of OD signals to polysaccharide concentrations [23].

### 2.8. Statistical Analysis

The sample size was determined by studying the power of a test of the hypothesis. A random sequence by the encoder was used to generate the random allocation of this study. All data were checked for normal distribution with the Kolmogorov-Smirnov test. Testing for significant differences was assessed by two-way ANOVA using Tukey’s post-hoc test for pairwise comparisons (*p* < 0.05). Statistical analyses were performed by SPSS 19.0 (SPSS, Chicago, IL, USA).

## 3. Results and Discussions

**Figure 1:**
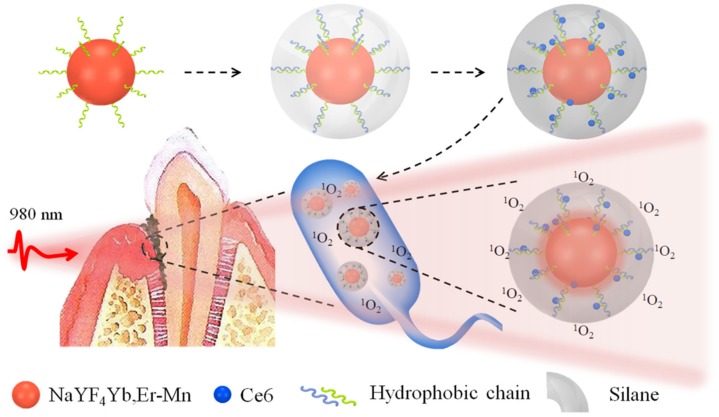
The schematic diagram of the composite structure.Ce6 and NaYF_4_:Yb,Er UCNPs were combined with amphiphilic silane modification through hydrophobic-hydrophobic interaction. Hydrophobic side chain of the silane (blue) and hydrophobic group (green) on the surface of NaYF_4_:Yb,Er UCNPs can attach each other forming the close coating layer. After entering into the bacteria in the area of periodontal disease, singlet oxygen (^1^O_2_) can be effectively produced under the irradiation of 980 nm irradiation as shown in the figure. Mn doping greatly enhances the red emission and then the aPDT function.

The schematic Figure 1 illustrates the synthesis of UCNPs@Ce6@silane composites. The Ce6 molecules have hydrophobic nature and are difficult to be introduced inside the in vivo environment. In this case, the Ce6 molecules were loaded and coated with a very thin silane layer with a thickness of 2–3 nm. When the upconversion nanocomposite is endocytosed by bacterial, the UCNPs can emit green and red light under the excitation of 980 nm NIR light, and the Ce6 molecules within the hydrophobic layer can be excited by the upconversion red emission, performing the aPDT function. The singlet oxygen is highly cytotoxic and can efficiently damage a variety of biomolecules, such as protein, nucleic acids and lipids, and in this design the Ce6 molecules can be well encapsulated and triggered only in the infectious area.

**Figure 2:**
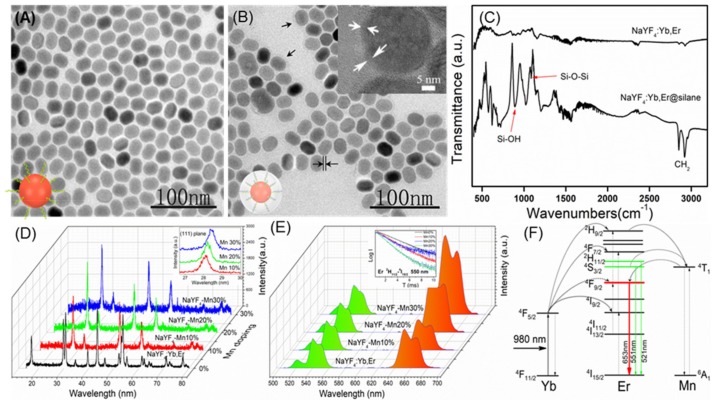
Characterization of the NaYF_4_:Yb^3+^,Er^3+^,Mn^2+^ (0%, 10%, 20%, 30%)UCNPs. (**A**) and (**B**) are the TEM images of NaYF_4_:Yb^3+^,Er^3+^ and silane modified NaYF_4_:Yb^3+^,Er^3+^UCNPs, respectively. Color insets in (**A**,**B**) showed the schematic structures of nanoparticles. HR-TEM insets in B showed the high magnification of single nanoparticles. (**C**) FT-IR spectra of NaYF_4_:Yb^3+^,Er^3+^ and silane modified NaYF_4_:Yb^3+^,Er^3+^UCNPs. (**D**) XRD pattern of NaYF_4_:Yb^3+^,Er^3+^,Mn^2+^ (0%, 10%, 20%, 30%) UCNPs. The inset is the amplified diffraction peak of 111 plane of the NaYF_4_:Yb^3+^,Er^3+^,Mn^2+^ (10%, 20%, 30%) UCNPs. (**E**) Upconversion fluorescence spectra of NaYF_4_:Yb^3+^,Er^3+^,Mn^2+^ (0%, 10%, 20%, 30%) UCNPs with 980 nm excitation. The inset is the lifetime of the ^2^H_11/2_ energy level corresponding to green emission. (**F**) Proposed energy transfer mechanisms under the excitation of 980 nm NIR.

As shown in Figure 2A,B the synthesized UCNPs and the silane modified UCNPs were characterized by TEM. It can be seen that the size distribution of both NPs is very uniform, about 25 nm for UCNPs and 30 nm for silane modified UCNPs. After silane coating, a thin layer of silane can be seen on the surface of the particles with a thickness of about 2–3 nm, as the black arrows point in Figure 2B. When a single nanoparticle is selected and characterized by high-power transmission electron microscopy, the thin layer of silane on the surface can be clearly examined, as indicated by the white arrow. The FTIR spectra before and after silane coating were shown in Figure 2C. It can be seen that, after silane modification with 18 carbon atoms chain, the characteristic peaks of Si-O-Si and Si-OH appear new compared to the unmodified UCNPs, indicating the successful modification of silane. Moreover, the intensity of the characteristic peak of -CH_2_ increases relative to the unmodified UCNPs due to the long carbon chain, which is also consistent with the experimental conditions. The zeta potential of NaYF_4_:Yb^3+^,Er^3+^@Ce6@silane and samples with the Mn doping percent of 10%, 20% and 30% composites are −28.5 mV, −27.8 mV, −33.5 mV, and −32.8 mV, respectively, indicating the high water solubility and stability.

It should be noted that, in addition to the complexity of the oral structure, the main difficulty of this NIR triggered PDT system lies in the design of the upconversion material. In some previous construction of photodynamic systems, photosensitizers were mostly supported on the nanocarriers by physical adsorption. For example, photosensitizer zinc phthalocyanine can be loaded into the mesopores of UCNP@mSiO_2_ NPs [19]. Though efficient production of ^1^O_2_ was realized after these photosensitizers were loaded by physical adsorption, it still faces serious leakage problems. Therefore, the FRET efficiency becomes low in the PDT process, causing systemic toxic side effects as well. In this coating strategy, by focusing on improving the efficiency of photodynamic therapy, the UCNPs were modified with silane, achieving water solubility and biocompatibility. In addition, the stable and very thin coating layer can well encapsulate the Ce6 molecules by hydrophobic-hydrophobic interaction and the followed hydrolysis of the silane further form the close composite, avoiding the Ce6 leakage. Silanes of different carbon chain lengths were also tested. Considering the higher loading amount of Ce6 molecules, silane with 18 C atoms was employed as the coating layer.

The maximum amount of Ce6 molecules that can be loaded in the hydrophobic layer was investigated. Here, by fixing the amount of UCNPs and silane, the content of Ce6 was changed to test the stability. Herein the mass of the UCNPs was fixed at 8 mg, and the corresponding mass of Ce6 were changed from 300 to 1000 μg. In this case, the content of Ce6 molecules was not to exceed 800 μg or precipitation would occur, producing a flocculent precipitate due to the leakage of the hydrophobic molecules (data not shown).

The structure of UCNPs with and without Mn doping were measured by XRD as shown in Figure 2D. Results confirmed the pure hexagonal phase NaYF_4_ without Mn doping. After the Mn ions were introduced into the nanocrystals, the crystal structure of the UCNPs changes from a hexagonal to a cubic phase. In addition, as the Mn ions doping increased, it could be found that the diffraction peak of 111 plane shifted toward the large angle direction (shown in inset), further illustrating the success of Mn ions doping. Note that the Mn doping do not influence the morphology and size of the UCNPs.

The upconversion luminescent property of silane-coated UCNPs and Mn-doped UCNPs were investigated. Upconversion luminescence were obtained based on the anti-Stokes mechanism. The spectra of different Mn-doped samples were excited using a 980 nm continuous diode laser, where the laser power was adjusted to 1 W. From the upconversion emission spectra in Figure 2E, the green emission was located at 528 and 546 nm, and red emission was located at 660 nm, corresponding to ^2^H_11/2_, ^4^S_3/2_ and ^4^F_9/2_ excited states to the ground state ^4^I_15/2_ transition of Er^3+^, respectively. Yb^3+^ ions serve as sensitizers which can absorb 980 nm photons more efficiently and then transfer energy to the activator Er^3+^, thus completing the upconversion green and red emissions. Different Mn doped samples showed no change of the peak position in the emission spectrum, but the ratios of the green/red emissions, which is dependent on the Mn doping amount. The existence of Mn^2+^ ions greatly influence the transition possibilities between green and red emissions of Er^3+^. As the Mn doping content increases from 0 to 30%, the proportion of red light gradually increases. The fine-tuning of red/green emissions could be attributed to nonradiative energy transfer from the ^2^H_9/2_ and ^4^S_3/2_ levels of Er^3+^ to the ^4^T_1_ level of Mn^2+^, followed by back-energy transfer to the ^4^F_9/2_ level of Er^3+^ as shown in Figure 2F, resulting in an enhanced red emission output by rational controlling the Mn^2+^ doping level. The energy transfer from level ^4^S_3/2_ to the Mn ion can be proved by the lifetime since the occurrence of such non-radiative transitions will reduce lifetime value. Relative to the NaYF_4_:Yb^3+^,Er^3+^@Ce6@silane UCNPs, all of the Mn doped UCNPs show the decreased lifetime in the green emission energy level. In addition, as the Mn doping content increases, the lifetime decreases gradually, and due to that the non-radiative transition speed is much faster than the radiation transition, as shown in the inset in Figure 2E, indicating that the more efficient energy transfer to the Mn ion happens, thus causing the further enhancement of red emission. It should be noted that this luminescence property is beneficial for the Ce6 based aPDT, because the excitation of the Ce6 molecule is located in the red region, and, in addition, the Ce6 molecule is on the surface of the UCNPs with a very close distance, thus facilitating the upconversion aPDT.

**Figure 3:**
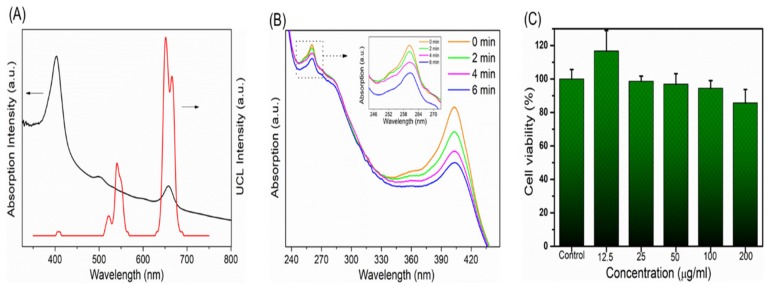
(**A**) Absorption spectrum and the upconversion spectrum of NaYF_4_:Yb^3+^,Er^3+^@Ce6@silane. Red emission region overlaps the absorption band of Ce6 molecules. (**B**) Generation of singlet oxygen generation under 980 nm irradiation. (**C**) Dark cytotoxicity of NaYF_4_:Yb^3+^,Er^3+^@Ce6@silane NPs on mouse fibroblast cell line L929: Viability of L929 cells vs. concentration of NaYF_4_:Yb^3+^,Er^3+^@Ce6@silane NPs after 24 h without irradiation in the dark (mean ± sd).

The absorption spectrum of the Ce6 molecule inside the composites and the upconversion fluorescence spectrum of Mn 30% doped UCNPs are shown in Figure 3A. The absorption at the red region is the primary excitation band of the Ce6 molecule for singlet oxygen production and this region is totally overlapped with the upconversion red emission band of its UCNPs carrier. Therefore, the enhanced upconversion red emission can further improve the aPDT effect. In addition, since the Ce6 molecule is located in the hydrophilic thin layer of the NPs, this energy transfer is very efficient for stimulating the PDT function. In the present study, up to 30% Mn was selected to dope into UCNPs to realize the enhancement of red light emission. Too much foreign element doping would lead to the change of crystal lattice, following with the functional variation of UCNPs. Besides, in this doping range (10–30%), the fluorescence intensity of red light increases with Mn doping, while the overall fluorescence intensity also decreases as the Mn content further increases. Previous studies have demonstrated that the introduction of sufficient Mn^2+^ ions into NaYF_4_:Yb/Er leads to a brilliant red emission, which is brighter by as much as 15 folds than that of Mn-free sample [25]. Therefore, in this doping scale, the highest red emission intensity was obtained, which is better for the efficacy of aPDT. In addition, in such doping situation, green light was also retained, which can be further used for fluorescence imaging. Currently, the up-conversion green light as imaging signal and red light as PDT treatment source need to be further investigated.

The upconversion PDT function of composite nanomaterials was tested using the singlet oxygen probe ABDA 9,10-fluorenyl-di(methylene)dimalonic acid [26]. Figure 3B shows the absorption spectra of magnetic nanocomposites under red light with an interval of 2 min. As the irradiation time increases, the absorption value of ABDA at 260 nm is reduced, indicating the generation of singlet oxygen. Furthermore, the absorption band at 400 nm showed the similar tendency, because the Ce6 molecules can also be consumed via the photodegradation of red light. It should be noted here that the detection efficiency of singlet oxygen is not very high according to the probe absorption because of the fact that the 980 nm excitation area is usually small, while the diffusion region of singlet oxygen is relatively large in the solution. This test proves that the composite material can produce singlet oxygen, indicating the successful material design and preparation. For periodontal disease treatments, the small 980 nm laser irradiation area is enough for sterilization. Dark cytotoxicity of NaYF_4_:Yb^3+^,Er^3+^@Ce6@silane NPs was investigated with L929 mouse fibroblast cells by CCK-8 assay, as shown in Figure 3C. The UCNPs show very good biocompatibility. The results showed that the cells were still 90% viable with the concentration of 200 μg/mL, indicating very low cytotoxicity. In this study, it should be attributed to the silane modification and the negatively-charged surface which could reduce cytotoxicity, showing great potential for new photosensitizer carriers in dental application.

**Figure 4:**
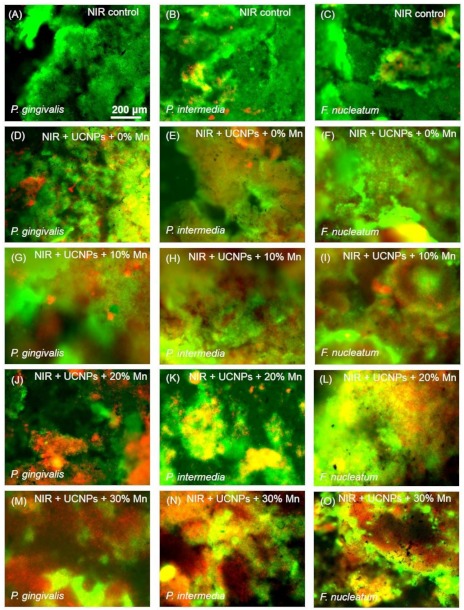
Images of live/dead cells of 4-day biofilms of three of *P. gingivalis* (left column), *P. intermedia* (middle column) and *F. nucleatum* (right column) on dentin squares for (**A**–**C**) NIR control, (**D**–**F**) NIR + NaYF_4_@Ce6@silane, (**G**–**I**) NIR + NaYF_4_-Mn10%@Ce6@silane, (**J**–**L**) NIR + NaYF_4_-Mn20%@Ce6@silane, and (**M**–**O**) NIR + NaYF_4_-Mn30%@Ce6@silane. All images had the same scale bar as shown in (**A**). Live bacteria were stained green. Bacteria with compromised membranes were stained red. Live and dead bacteria together or on the top of each other yielded yellow/orange colors. UCNPs with increasing Mn doping content showed more red staining of compromised bacteria under NIR (980 nm) irradiation.

Upconversion red light triggered PDT was first tested within biofilm experiments. The sample was irradiated with a 980 nm continuous diode laser which was adjusted to 750 J·cm^−2^ by tuning irradiation area for 3 min. Representative results of the live/dead analysis were performed and the results are shown in Figure 4 *P. gingivalis, P. intermedia* and *F. nucleatum* were selected as the bacteriostatic models in this work simulating early, middle and late stages of biofilm development, respectively, and colonized in plaque biofilms [27]. Live bacteria were stained as green which mainly in the control group and dead bacteria were stained red. In all three kinds of bacteria, NaYF_4_@Ce6@silane plays an efficient role in aPDT function. There are more and more dead bacteria in the groups with NaYF_4_@Ce6@silane and Mn doped NaYF_4_@Ce6@silane NPs under 980 nm light irradiation. The red color increases as the Mn doping increases, due to the enhanced upconversion red emission. The corresponding enhanced aPDT from Ce6 caused more and more dead bacteria. Note that the power density of the laser used in this study is strong enough for application of cell level in vitro. In addition, though the proposed periodontal bacteria locate in deepest periodontal pockets, usually 5–8 mm from the gingival margins, the power of the irradiation laser can still reach the threshold value. It is reported that the 980 nm laser can penetrate more than 0.7 cm in pork tissue without obvious reduction of power density [17,19,28].

Several kinds of UCNPs were applied to covert NIR to visible light or UV light for triggering PDT in tumor therapy or antibacterial application [29,30,31]. Gulzar et al. synthesized a nanocomposite based on nanographene oxide-UCNPs-Ce6 as a theranostic platform for the upconversion luminescence imaging-guided PDT/PTT of cancer [29]. The tremendous surface area of graphene oxide was allowed to house Ce6, as well as UCNPs. Remarkably, both the imaging and dual-mode treatments in this nanoplatform are stimulated by light, which unveils outstanding gains in terms of augmenting cancer killing specificity and decreasing side effects [29]. Numerous UCNPs-based nanomaterials with varieties of structures for photodynamic therapy in cancer treatment were summarized in a recent study [32]. On the other hand, for antibacterial application, Zhang et al. developed a photosensitizer (β-carboxyphthalocyanine zinc, CPZ) delivery system with UCNPs (LiYF_4_:Yb/Er) and polyvinylpyrrolidone (PVP) [30]. Such a near-infrared (NIR) triggered UCNPs-CPZ-PVP system significantly reduced the aggregation of CPZ and presented a high anti-infectious activity against multi-drug resistant bacteria (methicillin-resistant *Staphylococcus aureus* by 4.7 log and multi-drug resistant *Escherichia coli* by 2.1 log). Another study investigated the dual antibacterial behavior induced by the curcumin-UCNPs itself and induced by photodynamic therapy were demonstrated [31]. The results showed that nearly 100% methicillin-resistant *Staphylococcus aureus* was eradicated using curcumin-UCNPs under the NIR irradiation. However, to the best of our knowledge, the present study is the first report on application of near-infrared light to achieve photodynamic therapy for periodontitis treatment. We combine the upconversion luminescent material and the photosensitizer so that the near-infrared light with high tissue penetration depth can be utilized. More importantly, traditional aPDT had a minimal effect on the viability of microorganisms organized in a bacterial biofilm, which was probably due to the hydrophobic nature of the most photosensitizer molecules, leading to the reduced penetration of the photosensitizer into the biofilm matrix. The present study developed a silane coating as the shell of the UCNPs and embedded Ce6 inside the thin layer. This design would improve the hydrophilicity of the nanoparticles, and thus overcome the drainage of gingival crevicular fluid and high saliva fluid turnover [24]. Furthermore, the energy transfer would be more efficient for aPDT triggering due to the existence of Ce6 in the hydrophilic thin layer of the NPs. Therefore, this is a new exploration for the treatment of oral periodontitis, which is of great significance.

There are two possible aPDT mechanisms which can be described as follows: The triplet state photosensitizer Ce6 either can undergo a type I reaction, or a type II reaction. Type I: excited triplet Ce6 reacts directly with the macromolecule (protein, nucleic acid, lipids, etc.), generating free radicals or free radical ions by electron transfer, and further react with oxygen molecules, forming highly reactive oxides such as hydroxyl radicals, peroxides, etc. Type II: triplet photosensitizer Ce6 react with surrounding ground state oxygen molecules, generating singlet oxygen, which has ultrahigh cytotoxicity by oxidation and peroxidation of the cellular structure, microbial attack, destruction of the cell wall, and membrane system damage, thus affecting microbial metabolism and leading to cell death [33,34].

In this work, NaYF_4_@Ce6@silane nanocrystals release singlet oxygen through a type II reaction, which can penetrate into plaque and subsequently kill bacteria. Note that the nanosized material can enter the microorganisms by endocytosis which has been proven in previous studies [35,36,37]. In the present study, the high permeability of silane modified UCNPs are responsible for the efficient reactive oxygen generation and PDT effect.

Regarding the periodontitis treatment, aPDT that produces ROS possibly would not exacerbate the inflammatory response due to the following reasons: First, the role of PDT in inflammation is a complex process. Indeed, in tumor therapy, PDT could produce a strong inflammatory response of infiltration with neutrophils, mast cells, lymphocytes, monocytes, and macrophages [38]. However, PDT could also increase the stability of interleukin 10 (IL-10) RNA and/or increase the transcription efficiency of IL-10, which is an anti-inflammatory cytokine that inhibited cell-mediated immune responses [39]. Gollnick *et al* [40] reported that PDT could change the activity of a gene promoter and increase the expression level of IL-10. Therefore, these two processes may co-exist in the PDT therapy. Second, in general, ^1^O_2_ diffusion distance is only about 100 nm, and the half-life is <0.04 μs [41], the photosensitizer should be precisely delivered to the area of periodontal disease. Hence, the distance of ^1^O_2_ diffusion to the bacterial cells is of significant importance for the aPDT activity. Henderson et al. proposed that the ^1^O_2_-induced photodamage from porphyrin activation is usually localized to within 0.1 µm of its site of release [42]. Therefore, when the sterilization process is completed, the energy will disappear and the inflammation promotion effect on normal tissues is limited. These should be the main reasons for the efficient antibacterial properties, but without obvious inflammation. Alternatively, it is a promising approach to incorporation of anti-inflammatory agents (such as ceria, etc.) into the design of nanoparticles for aPDT application to reduce the potential risks in the future [43]. Considering the complicated oral structure and infectious area is always in deep tissue, these 980 nm laser excited UCNPs with large penetration depth are the most suitable photosensitizer carriers and energy transfer donors for antibacterial application. In addition, the Ce6 molecules are located on the surface of the UCNPs within very close distance because of the very thin silane-modified layer, forming the very close excitation distance. Therefore, besides the enhanced upconversion red light emission from NaYF_4_ UCNPs, efficient ^1^O_2_ production can be obtained for the antibacterial action against periodontal pathogens.

**Figure 5:**
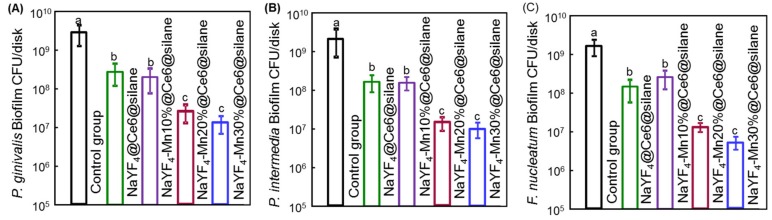
CFU counts of 4-day biofilms of (**A**) *P. gingivalis*, (**B**) *P. intermedia* and (**C**) *F. nucleatum* after PDT application (mean ± sd; *n* = 6). Nanoparticles with Mn doping would significantly decrease the CFU counts of all three-species biofilms. Note the log scale for the y-axis. Dissimilar letters indicated significant differences between each group (*p* < 0.05).

The CFU assay is the most essential for evaluating a new antimicrobial method. In this work, the CFU of 4-day biofilms of (A) *P. gingivalis*, (B) *P. intermedia* and (C) *F. nucleatum* after aPDT were measured and shown in Figure 5. In aPDT treatments, the biofilm matrix was easily disrupted with a deeper penetrated infrared light. The value for single-strain biofilms on dentin is different among the samples treated with different Mn doped NaYF_4_@Ce6@silane NPs. The control group shows the highest CFU value. After the treatment with the NPs and the 980 nm irradiation, the CFU experienced significant reductions for all three species compared to the control groups (*p* < 0.05). Among different bacterial species, NaYF_4_@Ce6@silane and NaYF_4_-Mn10%@Ce6@silane groups show similar CFU results, while, as Mn doping increases, the CFU values further decreases. A similar trend was observed among all the three bacterial species with the same aPDT procedure, indicating the universality of this upconversion aPDT agent. The three bacterial CFU counts of 4-day biofilms all showed a logarithmic reduction of more than 2 log with the Mn30% doped NPs. This high efficacy against periodontitis-related biofilms should be attributed to the high hydrophilic surface after silane modification, as well as the upconversion luminescence triggered aPDT.

It is known that, relative to planktonic bacteria, biofilms are much more difficult to kill because of the formation of extracellular polymeric substance inside the biofilm which greatly resist the entry of conventional antibacterial agents [44]. In such a situation, the singlet oxygen from PDT can play an important function due to its efficient diffusion and the oxidation of amino acids and DNA damage, while the aPDT agent carrier should be located in part of the disease. In this work, we further involved the upconversion aPDT with deep tissue penetration, which can solve the problem of bacterial treatment in deep periodontal tissue. 

**Figure 6:**
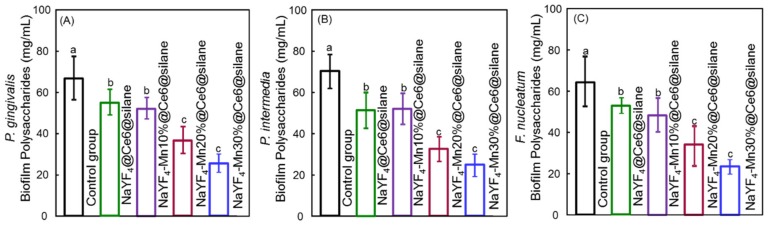
Polysaccharide production by 4-day biofilms of (**A**) *P. gingivalis,* (**B**) *P. intermedia* and (**C**) *F. nucleatum* on dentin squares (mean ± sd; *n* = 6). Dissimilar letters indicated significant differences between each group (*p* < 0.05).

The polysaccharide production of the biofilms was measured because polysaccharide is produced by live bacteria and then related to bacterial viability. Extracellular polymeric substance (EPS) protects pathogens from antibacterial agents and contributes to the virulence and pathogenicity of pathogens via small molecule mediated inter- and intra- species crosstalk. It is mainly composed of polysaccharides, proteins and extracellular DNA and accounts for about 90% of the total mass of the biofilm [45]. EPS with polysaccharide can be regulated by external stimuli since most glycoproteins are located on the outer membrane of Gram-negative bacteria. The polysaccharide production results of biofilms of (A) *P. gingivalis*, (B) *P. intermedia* and (C) *F. nucleatum* on dentin squares are plotted in Figure 6. For each species, the control group without UCNPs shows similar polysaccharide amounts (*p* > 0.1). The polysaccharide production for all three species was greatly reduced with the addition of NaYF_4_-Mn@Ce6@silane NPs under 980 nm irradiation compared to the control group (*p* < 0.05). These would probably be co-related to the decreased number of bacteria after aPDT treatments. Also, since the increased Mn doping enhanced the upconversion red emission and then aPDT function, the polysaccharide production decreased with the increasing Mn doping concentration from 0% to 30%. Therefore, the reduction in EPS via aPDT could reduce/destroy the protection of all three species of the bacteria.

Clinically, the subgingival biofilm is an aggregation of multispecies bacteria. Multispecies biofilms are more challenging to eradicate than single species biofilms and planktonic bacteria [46]. The early attached dominant species of bacteria are streptococci and members of the yellow and purple complexes, such as *Actinomycess* pp. which soon develop a polymicrobial community [47]. However, most studies also have been done in planktonic or single-species biofilms without regard for the complex microbial and biochemical changes occurring simultaneously [24]. Besides the standardized simple model for culturing in vitro, intact single-species biofilms were easy to realize in a normal laboratory. Within the limitations of this in vitro study, the biofilm model gave a simple means of determining the antimicrobial efficacy of novel nanocomposite with Mn doping upon NIR irradiation against three key periodontal pathogens. This methodology may be more clinically representative than the methods, which do not consider the microorganism in biofilms [48]. However, it still does not reproduce what happens clinically in the periodontal pocket. In such an environment, several mechanisms allow the growth and selection of several microorganisms, even after the treatment. Therefore, further studies should focus on the susceptibility of novel nanocomposite based aPDT against the multispecies biofilms and confirm the effects in animal studies.

## 4. Conclusions

NaYF_4_@Ce6@silane NPs with upconversion triggered aPDT function were designed in the treatment of periodontal disease. This upconversion fluorescent nanomaterial can overcome the limited tissue penetration depth of visible light in conventional PDT due to the infrared light excitation. The efficient photosensitizer Ce6 molecules were combined with NaYF_4_:Yb,Er NPs by amphiphilic silane method, through a hydrophobic-hydrophobic interaction between the hydrophobic side chain of the silane and the hydrophobic groups of the UCNPs, avoiding the leakage of the Ce6 molecules. With 980 nm NIR light excitation, the upconversion red emission can efficiently trigger the aPDT effect, because the primary excitation band of Ce6 molecules is totally overlapped with the upconversion red emission band of its UCNPs carrier, and, also, the Ce6 molecule is located in the hydrophobic thin layer of the NPs. Mn ions were doped into the UCNPs for the enhanced red emission and obtained improved energy transfer to Ce6 molecules for stimulating the aPDT function. The novel UCNPs had excellent biocompatibility low cytotoxicity with above 90% cell viability at the concentration of 200 μg/mL. By investigating the aPDT function on three bacterial of *P. gingivalis*, *P. intermedia* and *F. nucleatum*, an enhanced bacteriological therapeutic effect on bacterial and the corresponding biofilms was obtained with the increased Mn doping amount from 0 to 30%. Especially for Mn30% doped UCNPs, the three bacterial CFU counts of 4-day biofilms all showed a logarithmic reduction of more than 2 log. Moreover, Mn30% doped UCNPs had the lowest polysaccharide production among all groups. This upconversion aPDT design can overcome the problems of conventional PDT and has important application prospects in the treatment of periodontal diseases.

## Abbreviations

ANOVA	Analysis of variance
aPDT	Antibacterial photodynamic therapy
Ce6	Chlorin e6
CFU	Colony-forming units
CPW	Cysteine peptone water
EPS	Extracellular polymeric substance
FRET	Fluorescence resonance energy transfer
F. nucleatum	Fusobacterium nucleatum
P. gingivalis	Porphyromonas gingivalis
P. intermedia	Prevotella intermedia
ROS	Reactive oxygen species
UCNPs	Upconversion nanoparticles
XRD	X-ray diffraction

## References

[1] Ravald N., Johansson C.S. (2012). Tooth loss in periodontally treated patients. A long-term study of periodontal disease and root caries. J. Clin. Periodontol..

[2] Umeda M., Takeuchi Y., Noguchi K., Huang Y., Koshy G., Ishikawa I. (2004). Effects of nonsurgical periodontal therapy on the microbiota. Periodontolol. 2000.

[3] Pfitzner A., Sigusch B.W., Albrecht V., Glockmann E. (2004). Killing of periodontopathogenic bacteria by photodynamic therapy. J. Periodontol..

[4] Zhu H., Fang Y., Miao Q., Qi X., Ding D., Chen P., Pu K. (2017). Regulating near-infrared photodynamic properties of semiconducting polymer nanotheranostics for optimized cancer therapy. ACS Nano.

[5] Carrera E., Dias H., Corbi S., Marcantonio R., Bernardi A., Bagnato V.S., Hamblin M., Rastelli A.N.d.S. (2016). The application of antimicrobial photodynamic therapy, (aPDT) in dentistry: a critical review. Laser Phys..

[6] Haas R., Dörtbudak O., Mensdorff-pouilly N., Mailath G. (1997). Elimination of bacteria on different implant surfaces through photosensitization and soft laser. An in vitro study. Clin. Oral Implan. Res..

[7] Chan Y., Lai C.H. (2003). Bactericidal effects of different laser wavelengths on periodontopathic germs in photodynamic therapy. Laser. Med. Sci..

[8] Uekubo A., Hiratsuka K., Aoki A., Takeuchi Y., Abiko Y., Izumi Y. (2016). Effect of antimicrobial photodynamic therapy using rose bengal and blue light-emitting diode on Porphyromonas gingivalis in vitro: Influence of oxygen during treatment. Laser Ther..

[9] De Carvalho Goulart R., Thedei G., Souza S.L., Tedesco A.C., Ciancaglini P. (2010). Comparative study of methylene blue and erythrosine dyes employed in photodynamic therapy for inactivation of planktonic and biofilm-cultivated Aggregatibacter actinomycetemcomitans. Photomed. Laser Surg..

[10] Eick S., Markauskaite G., Nietzsche S., Laugisch O., Salvi G.E., Sculean A. (2013). Effect of photoactivated disinfection with a light-emitting diode on bacterial species and biofilms associated with periodontitis and peri-implantitis. Photodiagn. Photodyn..

[11] Street C.N., Pedigo L.A., Loebel N.G. (2010). Energy dose parameters affect antimicrobial photodynamic therapy–mediated eradication of periopathogenic biofilm and planktonic cultures. Photomed. Laser Surg..

[12] Dawson P., Romanowski M. (2018). Excitation modulation of upconversion nanoparticles for switch-like control of ultraviolet luminescence. J. Am. Chem. Soc..

[13] Cheng X., Ge H., Wei Y., Zhang K., Su W., Zhou J., Yin L., Zhan Q., Jing S., Huang L. (2018). Design for Brighter Photon Upconversion Emissions via Energy Level Overlap of Lanthanide Ions. ACS Nano.

[14] Würth C., Fischer S., Grauel B., Alivisatos A.P., Resch-Genger U. (2018). Quantum yields, surface quenching, and passivation efficiency for ultrasmall core/shell upconverting nanoparticles. J. Am. Chem. Soc..

[15] Zhu X., Su Q., Feng W., Li F. (2017). Anti-Stokes shift luminescent materials for bio-applications. Chem. Soc. Rev..

[16] Tsang M.K., Wong Y.T., Hao J. (2018). Cutting-Edge Nanomaterials for Advanced Multimodal Bioimaging Applications. Small Methods.

[17] Chen G., Ågren H., Ohulchanskyy T.Y., Prasad P.N. (2015). Light upconverting core–shell nanostructures: nanophotonic control for emerging applications. Chem. Soc. Rev..

[18] Dong H., Sun L.D., Yan C.H. (2015). Energy transfer in lanthanide upconversion studies for extended optical applications. Chem. Soc. Rev..

[19] Idris N.M., Jayakumar M.K.G., Bansal A., Zhang Y. (2015). Upconversion nanoparticles as versatile light nanotransducers for photoactivation applications. Chem. Soc. Rev..

[20] Chen B., Dong B., Wang J., Zhang S., Xu L., Yu W., Song H. (2013). Amphiphilic silane modified NaYF_4_: Yb, Er loaded with Eu (TTA)_3_ (TPPO)_2_ nanoparticles and their multi-functions: dual mode temperature sensing and cell imaging. Nanoscale.

[21] Li X., Liu X., Chevrier D.M., Qin X., Xie X., Song S., Zhang H., Zhang P., Liu X. (2015). Energy migration upconversion in manganese (ii)-doped nanoparticles. Angew. Chem. Int. Edit..

[22] Wang L., Xie X., Imazato S., Weir M.D., Reynolds M.A., Xu H.H. (2016). A protein-repellent and antibacterial nanocomposite for Class-V restorations to inhibit periodontitis-related pathogens. Mat. Sci. Eng. C-Mater..

[23] Fteita D., Könönen E., Söderling E., Gürsoy U.K. (2014). Effect of estradiol on planktonic growth, coaggregation, and biofilm formation of the Prevotella intermedia group bacteria. Anaerobe.

[24] Sun X., Wang L., Lynch C.D., Sun X., Li X., Qi M., Ma C., Li C., Dong B., Zhou Y. (2019). Nanoparticles having amphiphilic silane containing Chlorin e6 with strong anti-biofilm activity against periodontitis-related pathogens. J. Dent..

[25] Tian G., Gu Z., Zhou L., Yin W., Liu X., Yan L., Jin S., Ren W., Xing G., Li S. (2012). Mn^2+^ dopant-controlled synthesis of NaYF_4_:Yb/Er upconversion nanoparticles for in vivo imaging and Drug delivery. Adv. Mater..

[26] Wang X., Liu K., Yang G., Cheng L., He L., Liu Y., Li Y., Guo L., Liu Z. (2014). Near-infrared light triggered photodynamic therapy in combination with gene therapy using upconversion nanoparticles for effective cancer cell killing. Nanoscale.

[27] Wang L., Melo M.A.S., Weir M.D., Xie X., Reynolds M.A., Xu H.H. (2016). Novel bioactive nanocomposite for Class-V restorations to inhibit periodontitis-related pathogens. Dent. Mater..

[28] Sun X., Sun J., Dong B., Huang G., Zhang L., Zhou W., Lv J., Zhang X., Liu M., Xu L. (2019). Noninvasive temperature monitoring for dual-modal tumor therapy based on lanthanide-doped up-conversion nanocomposites. Biomaterials.

[29] Gulzar A., Xu J., Yang D., Xu L., He F., Gai S., Yang P. (2018). Nano-graphene oxide-UCNP-Ce6 covalently constructed nanocomposites for NIR-mediated bioimaging and PTT/PDT combinatorial therapy. Dalton T..

[30] Zhang Y., Huang P., Wang D., Chen J., Liu W., Hu P., Huang M., Chen X., Chen Z. (2018). Near-infrared-triggered antibacterial and antifungal photodynamic therapy based on lanthanide-doped upconversion nanoparticles. Nanoscale.

[31] Liu J., Yu M., Zeng G., Cao J., Wang Y., Ding T., Yang X., Sun K., Parviz J., Tian S. (2018). Dual antibacterial behavior of a curcumin–upconversion photodynamic nanosystem for efficient eradication of drug-resistant bacteria in a deep joint infection. J. Mater. Chem. B.

[32] Qiu H., Tan M., Ohulchanskyy T., Lovell J., Chen G. (2018). Recent progress in upconversion photodynamic therapy. Nanomaterials.

[33] Kashef N., Hamblin M.R. (2017). Can microbial cells develop resistance to oxidative stress in antimicrobial photodynamic inactivation?. Drug Resist. Update..

[34] Diogo P., Fernandes C., Caramelo F., Mota M., Miranda I.M., Faustino M., Neves M., Uliana M.P., de Oliveira K.T., Santos J.M. (2017). Antimicrobial photodynamic therapy against endodontic Enterococcus faecalis and Candida albicans mono and mixed biofilms in the presence of photosensitizers: A comparative study with classical endodontic irrigants. Front. Microbiol..

[35] Ranjan S., Ramalingam C. (2016). Titanium dioxide nanoparticles induce bacterial membrane rupture by reactive oxygen species generation. Environ. Chem. Lett..

[36] Huang L., Xuan Y., Koide Y., Zhiyentayev T., Tanaka M., Hamblin M.R. (2012). Type I and Type II mechanisms of antimicrobial photodynamic therapy: An in vitro study on gram-negative and gram-positive bacteria. Laser. Surg. Med..

[37] Maness P.C., Smolinski S., Blake D.M., Huang Z., Wolfrum E.J., Jacoby W.A. (1999). Bactericidal activity of photocatalytic TiO2 reaction: toward an understanding of its killing mechanism. Appl. Environ. Microbiol..

[38] Gollnick S.O., Lee B.Y., Vaughan L., Owczarczak B., Henderson B.W. (2001). Activation of the IL-10 Gene Promoter Following Photodynamic Therapy of Murine Keratinocytes. Photochem. Photobiol..

[39] Castano A.P., Mroz P., Hamblin M.R. (2006). Photodynamic therapy and anti-tumour immunity. Nat. Rev. Cancer.

[40] Gollnick S.O., Liu X., Owczarczak B., Musser D.A., Henderson B.W. (1997). Altered expression of interleukin 6 and interleukin 10 as a result of photodynamic therapy in vivo. Cancer Res..

[41] Misba L., Zaidi S., Khan A.U. (2018). Efficacy of photodynamic therapy against Streptococcus mutans biofilm: Role of singlet oxygen. J. Photoch. Photobio. B.

[42] Henderson B.W., Dougherty T.J. (1992). How does photodynamic therapy work?. Photochem. Photobiol..

[43] Li X., Qi M., Sun X., Weir M.D., Tay F.R., Oates T.W., Dong B., Zhou Y., Wang L., Xu H.H. (2019). Surface treatments on titanium implants via nanostructured ceria for antibacterial and anti-inflammatory capabilities. Acta Biomater..

[44] Wang L., Xie X., Qi M., Weir M.D., Reynolds M.A., Li C., Zhou C., Xu H.H. (2019). Effects of single species versus multispecies periodontal biofilms on the antibacterial efficacy of a novel bioactive Class-V nanocomposite. Dent. Mater..

[45] Li S.W., Sheng G.P., Cheng Y.Y., Yu H.Q. (2016). Redox properties of extracellular polymeric substances, (EPS) from electroactive bacteria. Sci. Rep..

[46] Barraud N., Storey M.V., Moore Z.P., Webb J.S., Rice S.A., Kjelleberg S. (2009). Nitric oxide-mediated dispersal in single-and multi-species biofilms of clinically and industrially relevant microorganisms. Microb. Biotechnol..

[47] Quirynen M., Vogels R., Peeters W., van Steenberghe D., Naert I., Haffajee A. (2006). Dynamics of initial subgingival colonization of ‘pristine’peri-implant pockets. Clin. Oral Implan. Res..

[48] Sena N., Gomes B., Vianna M., Berber V., Zaia A., Ferraz C., Souza-Filho F. (2006). In vitro antimicrobial activity of sodium hypochlorite and chlorhexidine against selected single-species biofilms. Int. Endod. J..

